# The Influence of Betulin and Its Derivatives on Selected Colorectal Cancer Cell Lines’ Viability and Their Antioxidant Systems

**DOI:** 10.3390/cells13161368

**Published:** 2024-08-17

**Authors:** Marcel Madej, Celina Kruszniewska-Rajs, Magdalena Kimsa-Dudek, Agnieszka Synowiec-Wojtarowicz, Elwira Chrobak, Ewa Bębenek, Stanisław Boryczka, Stanisław Głuszek, Jolanta Adamska, Sebastian Kubica, Jarosław Matykiewicz, Joanna Magdalena Gola

**Affiliations:** 1 Department of Molecular Biology, Faculty of Pharmaceutical Sciences in Sosnowiec, Medical University of Silesia, 40-055 Katowice, Poland; ckruszniewska@sum.edu.pl (C.K.-R.); jolaa@sum.edu.pl (J.A.); sebastian.kubica@sum.edu.pl (S.K.); 2 Department of Nutrigenomics and Bromatology, Faculty of Pharmaceutical Sciences in Sosnowiec, Medical University of Silesia, 40-055 Katowice, Poland; mkimsa@sum.edu.pl (M.K.-D.); asynowiec@sum.edu.pl (A.S.-W.); 3 Department of Organic Chemistry, Faculty of Pharmaceutical Sciences in Sosnowiec, Medical University of Silesia, 40-055 Katowice, Poland; echrobak@sum.edu.pl (E.C.); ebebenek@sum.edu.pl (E.B.); boryczka@sum.edu.pl (S.B.); 4 Department of Surgical Medicine with the Laboratory of Medical Genetics, Institute of Medical Sciences, Collegium Medicum, Jan Kochanowski University, 25-317 Kielce, Poland; sgluszek@wp.pl (S.G.); jaroslaw.matykiewicz@onkol.kielce.pl (J.M.); 5 Department of Clinic Oncological Surgery Holycross Center, 25-317 Kielce, Poland

**Keywords:** betulin, derivatives, oxidative stress, colorectal cancer, cell lines, CAT, SOD, GPx, synthesis, cytotoxicity

## Abstract

Oxidative stress is considered one of the main reasons for the development of colorectal cancer (CRC). Depending on the stage of the disease, variable activity of the main antioxidant enzymes, i.e., superoxide dismutase (SOD), catalase (CAT) and glutathione peroxidase (GPx), is observed. Due to limited treatment methods for CRC, new substances with potential antitumor activity targeting pathways related to oxidative stress are currently being sought, with substances of natural origin, including betulin, leading the way. The betulin molecule is chemically modified to obtain new derivatives with improved pharmacokinetic properties and higher biological activity. The aim of this study was to evaluate the effects of betulin and its new derivatives on viability and major antioxidant systems in colorectal cancer cell lines. The study showed that betulin and its derivative EB5 affect the antioxidant enzyme activity to varying degrees at both the protein and mRNA levels. The SW1116 cell line is more resistant to the tested compounds than RKO, which may be due to differences in the genetic and epigenetic profiles of these lines.

## 1. Introduction

Colorectal cancer (CRC) is one of the most common types of digestive tract cancer, ranking third in mortality worldwide [1,2]. The main risk factors closely linked to its development are lifestyle-related, i.e., low physical activity, a diet of highly processed food, smoking and others [1,2]. The occurrence of these cancers is only associated with genetic or hereditary predisposition to a small extent [1,2]. Despite relatively accurate diagnostic methods, treatment is still limited to surgical resection of part of the colon, the use of chemotherapeutic agents, e.g., 5-fluorouracil (5FU), cisplatin, etc., or a combination of the above. In some cases, immunotherapy is also possible [1,3]. Another problem with chemotherapy is the lack of a targeted mechanism of action on cancer cells specifically, causing damage to the patient’s normal cells as well [1,2,3]. Therefore, new therapeutic solutions are being sought in the field of cancer cell-targeted drug therapy, where substances of natural origin, such as galangin, kaempferol, curcumin or betulin, are leading the way [4,5].

Betulin (lup-20(29)-ene-3β,28-diol) (B) as a compound is classified as a lupane-type pentacyclic triterpene. It is one of the substances of natural origin, with the largest amounts occurring in the outer bark of the Betula species [5,6]. Pharmacologically, it has antiviral, anti-inflammatory, antioxidant and antitumor effects, which have been proven by several studies [5,7,8]. Nevertheless, due to its poor bioavailability, it is subjected to chemical modifications, thus improving its pharmacokinetic properties and obtaining new derivatives. This is possible due to the presence of three reactive groups at positions C-3, C-19 and C-28 in its structure [9].

One of the elements affecting the development of CRC is an imbalance between the production and elimination of reactive oxygen species (ROS) called oxidative stress [10,11]. In addition to this, the production of ROS is also influenced by environmental factors and in vitro maintenance of cell culture [11]. Two systems that remove reactive oxygen species exist: non-enzymatic systems, among which substances delivered to the body can be included, and enzymatic systems, among which superoxide dismutase (SOD), catalase (CAT) and glutathione peroxidase (GPx) can be distinguished [12,13]. The first of these removes the oxidative radicals to hydrogen peroxide and oxygen. It occurs in three isoforms, which differ in cellular localization. SOD1 is localized in the cytoplasm and SOD2 in the mitochondrion, while SOD3 occurs extracellularly [14,15,16,17]. Catalase, on the other hand, protects the cell from the toxic effects of a byproduct of cellular processes—hydrogen peroxide—by distributing it to water and molecular oxygen. GPx, in turn, is an enzyme that inactivates hydrogen peroxide and hydroperoxides of lipids. Reduced activity of this enzyme can cause an increase in oxidative stress, thereby affecting inflammatory reactions, which are among the factors that initiate the tumorigenic process leading to the development of CRC [18].

ROS can oxidize polyunsaturated fatty acids (PUFAs), resulting in the production of malondialdehyde (MDA) and 4-hydroxy-2-nonenal (HNE). The formed compounds, mainly HNE, may be involved in the development of colorectal cancer by affecting cyclooxygenase (COX), mainly COX-2, leading to activation of angiogenesis and inhibition of cell death, or through activation of the Wnt/β-catenin pathway [19]. MDA, in turn, stimulates DNA damage [11,19].

Betulin and its derivatives have been shown to induce apoptosis by decreasing the mitochondrial outer membrane potential (MOMP), resulting in the induction of ROS production and thus indirectly leading to the death of the cancer cells [20,21]. Therefore, the aim of this study was to evaluate the anticancer and antioxidant properties of betulin and its derivatives in six colorectal cancer cells with different malignancies and varying genetic and epigenetic profiles.

## 2. Materials and Methods

### 2.1. Synthesis of Selected Betulin Derivatives

Betulin was applied as substrate in the synthesis of derivatives EB5 (28-propynylobetulin) and ECH147 (29-diethoxyphosphoryl-28-propynylobetulin). Compound EB5 was obtained from betulin by the Steglich method using propynoic acid, DCC and DMAP [22]. A multistep synthetic path enabled the transformation of betulin into a phosphonate derivative ECH147 [23]. The compounds after purification by column chromatography were characterized by ^1^H and ^13^C NMR (Bruker AVANCE III HD 600, MA, USA), IR (IRAffinity-1 FTIR spectrometer; Shimadzu Corporation, Kyoto, Japan) and HRMS analysis (Bruker Impact II, Billerica, MA, USA). Spectroscopic data and melting points determined for compounds EB5 and ECH147 were consistent with values in the literature [22,23].

**Figure 1:**
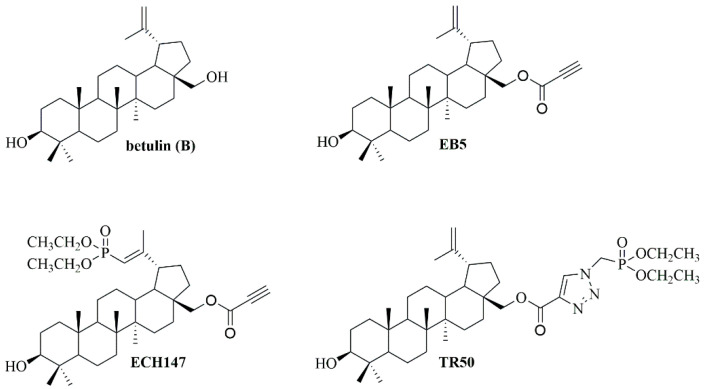
Chemical structure of betulin, EB5, ECH147 and TR50.

The cycloaddition reaction of the alkynyl derivative EB5 with diethyl (azidomethyl)phosphonate in toluene in the presence of copper(I) iodide led to the production of triazole TR50. The spectroscopic analysis of the TR50 (28-betulinyl (diethoxyphosphoryl)methyl)-1*H*-1,2,3-triazole-4-carboxylate) compound and the procedure for its preparation are included in the Supplementary Materials. Chemical structures of tested compounds are presented in Figure 1.

### 2.2. Culture Conditions for Six Colorectal Cancer Cell Lines and Normal Colonocytes

Six colorectal cancer cell lines, RKO, HT29, DLD-1, HCT116, Caco2 and SW1116 (CRL-2577, HTB-38, CCL-221, CCL-247, HTB-37, CCL-233; ATCC, Manassas, VA, USA, respectively, for CRC lines), and one normal cell line, CCD-841CoN (CRL-1790, ATCC, USA), were routinely cultured at 37 °C in a 5% CO_2_ incubator (Direct Heat CO_2_; Thermo Scientific, Waltham, MA, USA) in cell line-appropriate culture medium according to the manufacturer’s recommendations. Eagle’s minimum essential medium (EMEM) was used to culture RKO, Caco2 and CCD-841CoN lines, while McCoy’s 5A medium was used for HT29 and HCT116 lines. DLD-1 cells were grown in RPMI-1640 medium, whereas SW1116 cells were maintained in Leibovitz’s L-15 medium. All culture media were supplemented with 10% fetal bovine serum (EuroClone, Milan, Italy), and gentamycin at the concentration 50 mg/mL (BioWhittaker, Lonza, Basel, Switzerland) was also applied to prevent culture contamination.

### 2.3. Assessment of the Cytotoxicity of Tested Compounds

To evaluate the cytotoxicity of betulin, EB5, ECH147, TR50, cisplatin (CDDP) and 5FU on the tested cell lines, the MTT (3-[4,5-dimethylthiazol-2-yl]-2,5-diphenyltetrazolium bromide) assay (Sigma-Aldrich, St Louis, MO, USA) was used. For this purpose, cells were exposed to the test compound for 24 h at concentrations of 0.1, 1, 10, 20, 50 and 100 μg/mL. Control cells were not treated with the compounds. MTT (0.25 mg/mL) was then added to the cultures and incubated for 3 h at 37 °C. After a time, the cells were washed with phosphate buffered saline (PBS), and next, formazan dye was dissolved in 100 μL of dimethyl sulfoxide (Sigma-Aldrich, St Louis, MO, USA). Absorbance was measured at 540 nm using a Wallac 1420 VICTOR microplate reader (PerkinElmer, Waltham, MA, USA).

Based on the cytotoxicity results, betulin and its EB5 derivative at 10 µg/mL were selected for further analysis, due to the fact that even the highest concentration of EB5 did not reduce the viability of normal cells in the CCD-841CoN line below 50%. 5FU and cisplatin at the same concentration were used as reference compounds. In addition, two colorectal cancer cell lines, RKO and SW1116, were distinguished based on a different response to the EB5 derivative. To perform subsequent analyses, the selected CRC lines were treated with EB5 derivative at a concentration of 10 µg/mL for 24 h. Untreated cells were used as a negative control. Three biological replicates were performed.

### 2.4. Total Antioxidant Capacity of Tested Cells

To examine the antioxidant capacity of the cells as well as lipid peroxidation and to evaluate the activity of antioxidant enzymes, the cells were suspended in lysis buffer (containing 4 mg of SIGMAFAST™ Protease Inhibitor Cocktail and 10 µL of Phosphatase Inhibitor Cocktail 3 in 1 mL of PBS; Sigma-Aldrich, St Louis, MO, USA). Subsequently, the cells were placed in liquid nitrogen for 30 min, followed by storage at −80 °C until further analysis.

The ABTS+ radical cation was used to assess the total antioxidant capacity (TAC) of the tested cells. The principle of the method is based on the reduction of the formed blue-green chromophore ABTS+ as a result of the reaction of ABTS (Sigma-Aldrich, St Louis, MO, USA) with potassium persulphate (Sigma-Aldrich, St Louis, MO, USA). The degree of decolorization of the ABTS+ radical cation depends on the antioxidant activity of the cells and is determined as a function of concentration and time in relation to Trolox reactivity (TEAC-Trolox equivalent antioxidant capacity). The decrease in absorbance was measured at 734 nm using a Wallac 1420 VICTOR microplate reader (PerkinElmer, Waltham, MA, USA) [21,24].

### 2.5. Evaluation of Lipid Peroxidation Using MDA Assay

To assess the level of lipid peroxidation, the Malondialdehyde Assay Kit (Aoxre BioSciences, Burlingame, CA, USA) was used, according to the manufacturer’s recommendations. The reaction of the N-methyl-2-phenylindole (NPMI) reagent with MDA results in the formation of a carbocyanine dye, the absorbance of which was measured at 586 nm using a Wallac 1420 VICTOR microplate reader (PerkinElmer, Waltham, MA, USA).

### 2.6. Assessment of CAT, SOD and GPx Activity

Superoxide dismutase (SOD), glutathione peroxidase (GPx) and catalase (CAT) activities were assessed using a commercially purchased Cayman Chemicals Superoxide Dismutase Assay Kit, a Cayman Chemicals Glutathione Peroxidase Assay Kit and a Catalase Assay Kit (Cayman Chemicals, Ann Arbor, MI, USA), respectively, according to the manufacturer’s recommendations and based on a previous study [21]. The cell supernatant from previously prepared cell lysates of the tested lines provided material for further analyses. The level of change in absorbance was measured spectrophotometrically at the appropriate wavelength for the assay, i.e., for SOD in the range 440–460 nm, for GPx at 340 nm and for CAT at 540 nm.

### 2.7. Ribonucleic Acid Extraction from Tested Cells Treated With Compounds

Total RNA from treated cells was extracted using TRIzol reagent (Sigma-Aldrich, St Louis, MO, USA) according to the manufacturer’s instructions. The obtained RNA was assessed qualitatively and quantitatively by agarose gel electrophoresis and by spectrophotometric measurement using MaestroNano MN-913 (MaestroGen Inc., Las Vegas, NV, USA), respectively. The obtained extracts provided the starting material for the assessment of genes changes at the mRNA level.

### 2.8. Assessment of Changes in SOD1, SOD2, CAT and GPX3 Gene Expression at the mRNA Level

Changes in the expression of *SOD1*, *SOD2*, *CAT* and *GPX3* genes involved in antioxidant systems were assessed by real-time RT-qPCR using a LightCycler^®^ 480 System apparatus (Roche, Basel, Switzerland). A commercially purchased GoTaq^®^ 1-Step RT-qPCR System (Promega, Madison, WI, USA) was used, while the primers and reaction conditions were the same as in the previous study performed by Kruszniewska-Rajs et al. [21]. GAPDH was used as the reference gene. The mRNA expression level studied genes were then determined by the 2^−ΔCt^ relative quantification method. All samples were tested in triplicate. The specificity of the reaction was evaluated based on the melting temperature curve, as well as using electrophoresis of the amplification products in a 2% agarose gel.

### 2.9. Statistical Analysis

Statistical analysis was performed using STATISTICA 13.3 (TIBCO Software Inc., Palo Alto, CA, USA). All protein (biochemical) parameters were recalculated on 10^6^ cells. Statistically significant results were considered those with *p* < 0.05. Qualitative data were presented as mean ± standard deviation (SD) and presented graphically. Assessment of the type of data distribution was performed using the Shapiro–Wilk W test, while a one-way ANOVA test was used to compare quantitative variables between groups. Tukey’s post hoc test was also applied.

## 3. Results

### 3.1. Cytotoxicity of Tested Compounds

**Figure 2:**
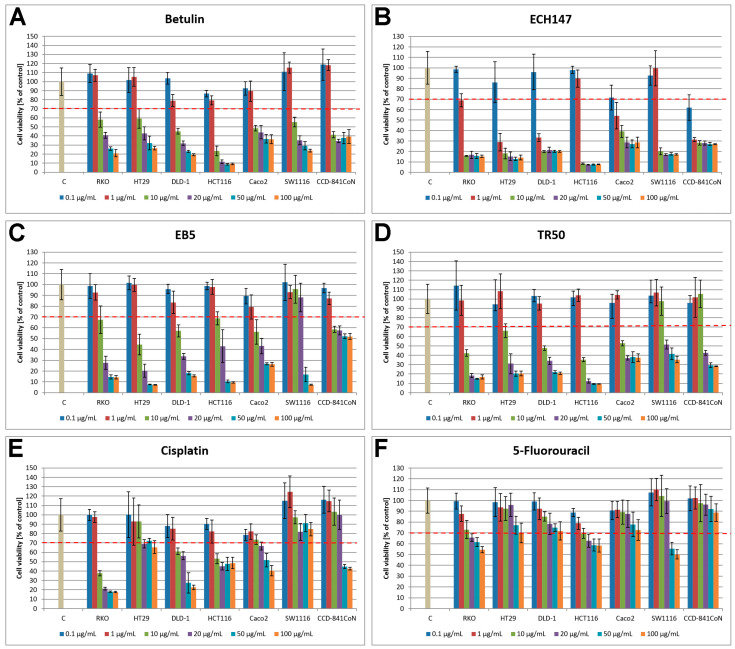
Cell viability of six colorectal cancer lines and normal cell line after treatment with betulin (**A**), ECH147 (**B**), EB5 (**C**), TR50 (**D**) derivatives, cisplatin (**E**) and 5-fluorouracil (**F**) with different concentrations. The dashed line indicates a 70% viability cut-off. C—100% viability for control calculated by averaging absorbance values for all cell lines not treated with compounds. Bars indicate percentage of viability relative to control; whiskers represent standard deviation (SD).

The obtained cytotoxicity results showed that the tested compounds at a concentration of 0.1 µg/mL, except the ECH147 derivative, did not cause a cytotoxic effect in each of the tested colorectal cancer cell lines, since the viability did not decrease below 70%. In addition, the effect is dependent on the cell lines studied and also the concentration of the compounds (Figure 2).

In the case of betulin (Figure 2A), the highest cytotoxicity was observed against the HCT116 cell line, which was higher than the CCD-841CoN cell line. Interestingly, only the RKO and SW1116 lines did not show a decrease in the viability of these cells of more than 50% after treatment with betulin at a concentration of 10 µg/mL.

For ECH147 derivative at concentrations higher than 1 µg/mL, a significant decrease in cell viability was observed in each of the tested cell lines. However, the HCT116 cell line showed higher sensitivity towards this derivative than control cells (Figure 2B).

The results obtained for the EB5 derivative show a high cytotoxic effect at concentrations of 50 µg/mL and 100 µg/mL compared to cells of the CCD-841CoN line (Figure 2C). Interestingly, the greatest effect of EB5 on viability was observed in line HT29 in comparison to betulin at the same concentration.

The TR50 derivative showed lack of sensitivity of SW1116 cells to this compound at a concentration of 10 µg/mL in comparison with other tested CRC cell lines and normal colonocytes (Figure 2D). The cytotoxicity effect of cisplatin (Figure 2E) and 5FU (Figure 2F) was weaker than that of the other compounds.

Due to the obtained results, two cell lines, RKO and SW1116, exhibiting different sensitivity to betulin, EB5 derivative, cisplatin and 5FU at a concentration of 10 µg/mL, were selected for studies at the protein and mRNA level. Additionally, they were selected due to the fact that their viability did not fall below 50%, with the exception of the RKO line treated with cisplatin at the same concentration.

In addition, the IC_50_ value was calculated for each of the studied cell lines and for the tested compounds, presented in Table 1.

### 3.2. Total Antioxidant Capacity of RKO and SW1116 Cell Lines

Betulin and its derivative EB5 affect the redox homeostasis of colorectal cancer cells of the RKO and SW1116 cell lines.

**Figure 3:**
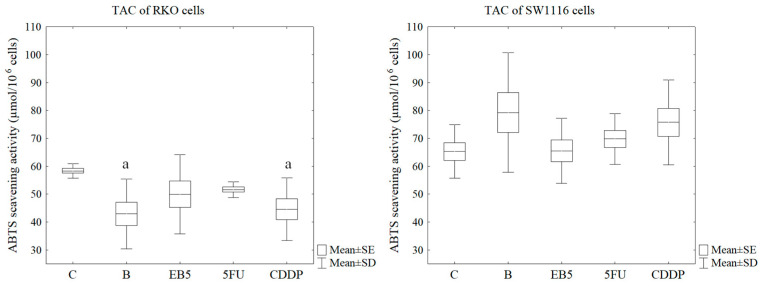
Total antioxidant capacity of RKO and SW1116 cell lines. Box and whisker plots present means ± standard error (SE) and standard deviation (SD); Tukey post hoc test (n = 9); C—control; B—cells treated with betulin; EB5—cells treated with EB5 derivative; 5FU—cells treated with 5-fluorouracil; CDDP—cells treated with cisplatin; a *p* < 0.05 vs. C.

Total antioxidant capacity (TAC) of cells can be considered as an indicator reflecting the level of oxidative stress. A reduction of about 26.4% in TAC was observed in RKO cells under the influence of betulin (B) compared to the control cells (Tukey post hoc test, *p* = 0.018). Cisplatin also had a similar effect—decrease in TAC by about 23.6% (Tukey post hoc test, *p* = 0.044). However, in SW1116 cells, no changes in total antioxidant capacity were observed under the influence of the tested compounds (Figure 3).

### 3.3. Level of Lipid Peroxidation

**Figure 4:**
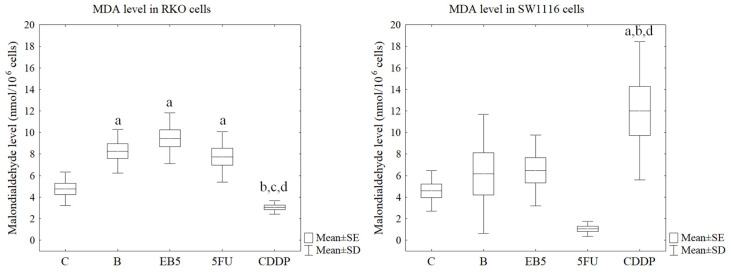
Level of lipid peroxidation in RKO and SW1116 cell lines. Box and whisker plots present means ± standard error (SE) and standard deviation (SD); Tukey post hoc test (n = 9); C—control; B—cells treated with betulin; EB5—cells treated with EB5 derivative; 5FU—cells treated with 5-fluorouracil; CDDP—cells treated with cisplatin; a *p* < 0.05 vs. C; b *p* < 0.05 vs. B; c *p* < 0.05 vs. EB5; d *p* < 0.05 vs. 5FU.

Malondialdehyde is an end product of the lipid peroxidation process. It can also be used as a biomarker of oxidative stress. Our studies revealed an increase in MDA levels in RKO cells treated with betulin (by about 73.1%), EB5 (by about 98.0%) and 5-fluorouracil (by about 62.2%) compared to the control cells (Tukey post hoc test, *p* = 0.003, *p* < 0.001, *p* = 0.016, respectively). In turn, cisplatin did not cause changes in MDA level compared to the control group. However, a higher level of MDA was observed in SW1116 cells after treatment with cisplatin compared to the control, betulin- and 5FU-treated cells (Tukey post hoc test, *p* = 0.0035, *p* = 0.049, *p* < 0.001, respectively). Nevertheless, betulin, EB5 and 5FU did not cause changes compared to the control (Figure 4).

### 3.4. Effect of the Studied Compounds on the Activity of the Antioxidant Enzymes SOD, GPx and CAT

**Figure 5:**
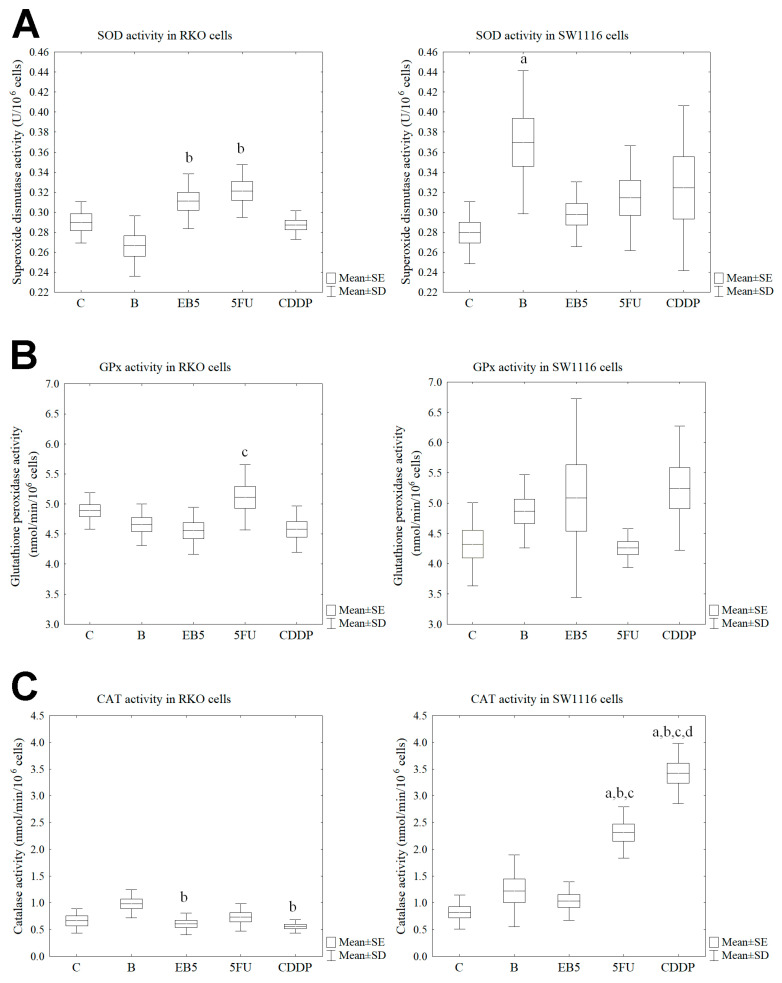
Evaluation of the activity of the SOD (**A**), GPx (**B**) and CAT (**C**) in RKO and SW1116 cell lines after treatment with the tested compounds. Box and whisker plots present means ± standard error (SE) and standard deviation (SD); Tukey post hoc test (n = 9); C—control; B—cells treated with betulin; EB5—cells treated with EB5 derivative; 5FU—cells treated with 5-fluorouracil; CDDP—cells treated with cisplatin; a *p* < 0.05 vs. C; b *p* < 0.05 vs. B; c *p* < 0.05 vs. EB5; d *p* < 0.05 vs. 5FU.

In the next stage, it was shown that the tested compounds slightly influence the activity of antioxidant enzymes in both cell lines. No changes in SOD activity were observed in RKO cells after treatment with the tested compounds compared to the control cells. However, a modest increase in SOD activity was noted in cells treated with EB5 and 5-fluorouracil compared to cells treated with betulin (Tukey post hoc test, *p* = 0.004, *p* < 0.001, respectively). In turn, in SW1116 cells, an increase in SOD activity (by about 32.3%) was observed only after betulin treatment compared to the untreated cells (Tukey post hoc test, *p* = 0.013) (Figure 5A). Moreover, GPx activity did not change compared to the control cells in both cell lines. A slight increase in its activity was only noted in RKO cells treated with 5FU vs. cells treated with EB5 (Tukey post hoc test, −0.042) (Figure 5B).

Also in the case of CAT activity in RKO cells, no changes were observed under the influence of treatment with the tested compounds compared to the control. A slight decrease in its activity was revealed in EB5- and CDDP-treated RKO cells compared to the betulin-treated cells (Tukey post hoc test, *p* = 0.008, *p* = 0.002, respectively). In turn, SW1116 cells were characterized by higher catalase activity after treatment with only 5FU and CDDP compared to the control (Tukey post hoc test, *p* < 0.001, *p* < 0.001, respectively), cells treated with betulin (Tukey post hoc test, *p* < 0.001, *p* < 0.001, respectively) and cells treated with EB5 (Tukey post hoc test, *p* < 0.001, *p* < 0.001, respectively). However, betulin and EB5 did not cause changes in its activity compared to the untreated cells (Figure 5C).

### 3.5. The Influence of Tested Compounds on mRNA Levels of SOD1, SOD2, CAT and GPX3 Genes

To evaluate expression changes in *SOD1*, *SOD2*, *CAT* and *GPX3* genes in RKO and SW1116 colorectal cancer cells exposed to the tested compounds, a real-time RT-qPCR analysis was performed.

The mRNA of the *GPX3* gene was not detected, both in untreated and treated RKO and SW1116 cell lines.

**Figure 6:**
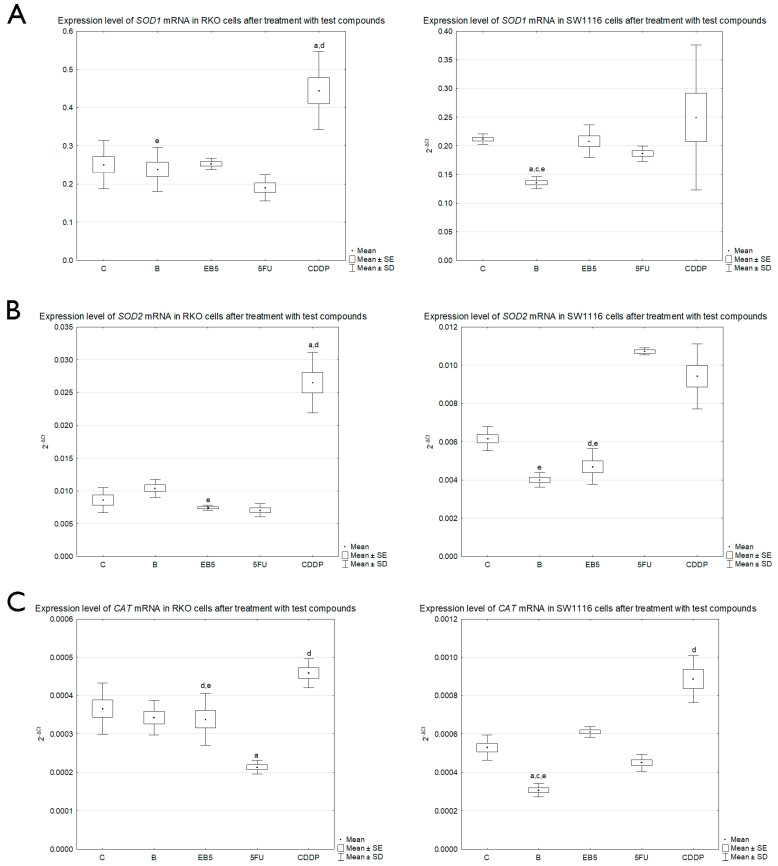
Effect of tested compounds on the expression levels of SOD1 (**A**), SOD2 (**B**) and CAT (**C**) genes in RKO and SW1116 cell lines. Box and whisker plots present means ± standard error (SE) and standard deviation (SD); ANOVA test; C—control; B—cells treated with betulin; EB5—cells treated with EB5 derivative; 5FU—cells treated with 5-fluorouracil; CDDP—cells treated with cisplatin; a *p* < 0.05 vs. C; b *p* < 0.05 vs. B; c *p* < 0.05 vs. EB5; d *p* < 0.05 vs. 5FU; e *p* < 0.05 vs. cisplatin.

The *SOD1* gene in RKO cells showed a statistically significant increase in expression between cisplatin vs. non-treated cells (*p* < 0.05), as well as between cisplatin and 5FU (*p* < 0.001). Additionally, comparing cells treated with betulin vs. cisplatin, a decrease in *SOD1* gene expression was observed (*p* < 0.01). In the case of the SW1116 line, a statistically significant difference in *SOD1* gene expression was shown between B vs. non-treated cells, B vs. EB5 derivative and B vs. cisplatin (*p* < 0.001, *p* < 0.05 and *p* < 0.05, respectively) (Figure 6A).

A statistically significant increase in *SOD2* gene expression between cisplatin-treated and untreated cells was noted in the RKO line (*p* < 0.05). Simultaneously, cisplatin-treated cells showed higher expression compared to those treated with 5FU (*p* < 0.001). Moreover, a significant decrease in *SOD2* gene expression between cells exposed to the EB5 derivative vs. cisplatin, *p* < 0.001, was also observed. The SW1116 colorectal cancer cell line showed a slightly different response to the tested compounds. Decreased *SOD2* gene expression in these cells was noted between cells treated with the derivative EB5 vs. cisplatin and EB5 vs. 5FU (*p* < 0.01 for both), as well as between B and cisplatin (*p* < 0.001) (Figure 6B). This may therefore suggest that chemotherapeutics cause an upregulation of SOD2 gene expression in SW1116 cells, while betulin and its derivative EB5 cause a decrease in the expression.

The expression level of the *CAT* gene, encoding catalase, was also analyzed (Figure 6C). RKO cells showed a significant decrease in expression between 5FU and untreated cells (*p* < 0.01), as well as between EB5 vs. cisplatin (*p* < 0.05). A significant increase in expression was observed between the EB5 vs. 5FU and cisplatin vs. 5FU (*p* < 0.05 and *p* < 0.001, respectively). No statistically significant changes in *CAT* gene expression were found between B and non-treated cells. Interestingly, a different expression profile of the *CAT* gene was observed in the SW1116 cell line after treatment with the tested compounds. A statistically significant decrease in *CAT* gene expression was observed in B-treated cells vs. untreated cells (*p* < 0.05). Moreover, the decrease in expression was higher in B vs. EB5 derivative and B vs. cisplatin (*p* < 0.001 for both). Furthermore, a difference in the expression between cisplatin and 5FU was also found (*p* < 0.001).

## 4. Discussion

Gastrointestinal cancers are one of the most frequent cancers worldwide among both men and women [1,5]. These include stomach cancer, esophageal cancer, hepatocellular carcinoma and colorectal cancer, which predominates in terms of incidence [1,5]. Inappropriate lifestyles, lack of physical activity, a diet rich in red processed meats and environmental factors increase the possibility of developing CRC [1,5]. Current diagnostics is quite accurate; however, treatment methods are limited and mainly involve the use of radiotherapy or chemotherapy, which is usually burdensome for the patient, or the resection of the part of the colon affected by the neoplastic lesion [1,5]. In some cases, immunotherapeutic agents based on monoclonal antibodies are used. However, new therapeutic solutions are still being sought in the field of medicinal substances with potential targeted antitumor activity, with compounds of natural origin, i.e., betulin, being the main focus [5,6]. Its proven broad spectrum of activity, including its potential antitumor properties, provides a valuable source for further research into new therapeutic substances for the treatment of CRC [5,6]. However, due to its relatively poor bioavailability and pharmacokinetic properties, it is undergoing chemical modifications to obtain new derivatives with improved biological and chemical features [5,6].

Undoubtedly, one of the key factors in the development of CRC is the disruption of the balance between the production and utilization of ROS by major antioxidant defense enzymes. It is assumed that excessive amounts of ROS can affect processes associated with DNA damage, as well as activate wall signaling pathways associated with carcinogenesis, proliferation and angiogenesis, leading to cell death [10,11]. With this in mind, it is possible to influence the inhibition of the tumorigenesis process by increasing the removal of high amounts of ROS in the cell, or, in the case of an already occurring neoplasm, to induce their increase in tumor cells. One of the key enzymes at the forefront of antioxidative protection are enzymes such as catalase, glutathione peroxidase and superoxide dismutase, which together are responsible for maintaining redox homeostasis in the cell [10,11].

The aim of the performed study was to evaluate cytotoxicity and influence on antioxidant status of betulin derivatives against six colorectal cancer lines and a normal colon cell line. Based on the results of the MTT test, the derivatives EB5 and ECH147 were chosen and the optimal concentration of the tested compounds, i.e., 10 µg/mL, was determined. Subsequently, two cell lines, RKO and SW1116, were selected for further studies due to the different response of these lines to the tested compounds. In order to evaluate the tested compounds on the antioxidant activity of the studied lines, the alteration in CAT, SOD and GPx enzyme activity was analyzed. Next, the level of lipid peroxidation and the total antioxidant capacity of the cells were evaluated after treatment with the tested compounds. The influence of these substances on the expression changes of *CAT*, *SOD1*, *SOD2* and *GPX3* genes at the mRNA level was also evaluated using a real-time RT-qPCR technique.

The MTT assay was used to study the cytotoxicity of the tested compounds towards colorectal cancer cells, according to which we observed differences in cell viability depending on the concentration and the tested compound. The strongest cytotoxic effect was observed for the ECH147 derivative regardless of the type of cell line. This may be due to the fact that this derivative is the only one of the tested ones which additionally has substituents at the C-29 position, which may increase its effectiveness towards cells; however, due to the fact that it also acts to the same extent on normal cells, it can be assumed that it does not show selectivity towards cancer cells. It also seems interesting that the EB5 derivative shows relatively greater cytotoxicity towards colorectal cancer cells compared to cisplatin. A similar study was carried out by Lubczyńska et al. [25, who evaluated the change in expression of genes related to the inflammatory process in the HT29 cell line after treatment with betulin and its alkynyl derivatives, including the EB5 derivative [25]. A viability assay using sulforhodamine B showed that CRC cells of the HT29 cell line were more sensitive to the effects of the EB5 derivative compared to cisplatin. In addition, they observed that the effect of this derivative was also weaker in comparison to betulin, which was also observed in our study [25].

Additionally, in the case of our study on other cell lines, we also noticed an interesting effect: SW1116 and RKO cell lines showed different sensitivity to the tested compounds. This may be related primarily to the degree of cancer from which they originate but also to the different genetic and epigenetic profile depending on the cell line [26]. The major differences that can affect this variable sensitivity are those dependent on mutations in the *BRAF* and *KRAS* genes. According to a study by Ahmed et al. [26,27] the RKO cell line has a V600E mutation in the BRAF gene, while the SW1116 cell line has a G12A mutation in the *KRAS* gene, which determines resistance to anti-EGFR treatment [26,27]. Therefore, it can be assumed that the ECH147 derivative, which showed a greater cytotoxic effect in the SW1116 cell line containing *KRAS^G12A^* mutations, may influence EGFR-related signaling pathways, leading to a better therapeutic effect than cisplatin or 5FU; however, further analysis is required. In addition, the best effect was observed at a concentration of each test compound of 10 µg/mL, which also served for further analysis.

In the next stage, the effect of the tested compounds in the RKO and SW1116 cell lines on the activity of enzymes involved in antioxidant defense was evaluated. The total antioxidant capacity of cells treated with the compounds was examined at the first step. In this case, significant differences were also noted. In RKO cells under the influence of betulin and cisplatin, a decrease in the TAC was observed, which may be related to the induction of stress in colorectal cancer cells [28,29]. For cisplatin, an increase in ROS production in cells through a decrease in antioxidant activity may result in an increase in cell sensitivity to cisplatin. Betulin showed a similar effect of decrease in TAC on the RKO cell line; however, to confirm the antioxidant and antitumor properties of betulin on selected CRC lines, further studies using an ROS inhibitor should be conducted [28,29]. In the case of the SW1116 cell line, no changes in the activity of the antioxidant system were observed. This difference may be due primarily to the previously mentioned genetic difference between the cell lines and their associated resistance to chemicals. Interestingly, no significant changes in the activity of antioxidant systems were observed for the derivatives tested. This may be due to the fact that the introduction of new substituents into the structure of betulin does not affect oxidative stress in the cancer cell. In addition, it is possible that intracellular components may interact with the derivatives causing their inactivation, thereby reducing the level of ROS in the cell [30].

In the second stage, the effect of oxidative stress induction on cellular particles was assessed by determining MDA. It is thought that malondialdehyde levels may be a marker of cell damage [31,32]. In our study, we observed an increase in MDA levels in RKO cells after treatment with all compounds except cisplatin, which did not significantly affect its pattern. This may be due to the genetically and epigenetically different nature of the tested line compared to the SW1116 cell line. Interestingly, the SW1116 cell line reacted differently, i.e., an increase in MDA levels was observed only after cisplatin treatment. Taking into account the fact that increased levels of lipid peroxidation in neoplastic cells can negatively affect these cells, this may suggest that the tested compounds could have a potential anticancer effect on the RKO cell line due to the fact that MDA forms adducts that affect cell signaling mainly related to proliferation and inflammation; however, more research should be performed [31].

Subsequently, the change in the main antioxidant enzymes, i.e., the activity of SOD, GPx and CAT in the cells, was also evaluated. Superoxide dismutase is considered the first line of antioxidant defense, an enzyme that catalyzes the superoxide anion radical to oxygen and hydrogen peroxide. It exists in three isoforms, SOD1, SOD2 and SOD3, differing in cellular localization. As a result of our study, we noticed differences in the activity of this enzyme depending on the cell line. The RKO line showed higher SOD activity after treatment with 5FU and an EB5 derivative. Interestingly, the alkynyl derivative significantly increased SOD activity, while its native form, betulin, decreased. Moreover, the opposite situation was observed in cells of SW1116 line, in which an increase in SOD activity was observed after treatment with betulin. A study conducted by Dubinin et al. showed that the mechanism of action of betulin and its derivatives may be based on an effect on the mitochondrial membrane and inhibit the mitochondrial respiratory chain complex, accompanied by an increase in ROS production [33,34]. In addition, different results were obtained by the team of Abd Al Moaty et al. [35], who showed on colon cancer cell lines that thiazolidinediones derivatives cause a decrease in SOD activity in cells of the Caco2 line, which contributes to an increase in ROS production in neoplastic cells and thus may cause death of neoplastic cells [35]. However, in our study, SOD levels were increased for the SW1116 line after betulin treatment, which may be the reason for the sudden release of oxidative stress, due to the fact of the 24h exposure time of the tested compounds [21]. Possibly, the decrease would be observable with longer exposure to the tested compounds, which needs to be verified in further studies. Nevertheless, betulin effectively acted on the RKO line, where it reduced SOD activity.

Glutathione peroxidase, on the other hand, acts as a second enzyme in the antioxidant system, and it is responsible for the removal of hydrogen peroxide and lipid peroxides [36]. In our study, we only observed an increased activity pattern of GPx in the RKO line after 5FU treatment, while no changes were observed in the SW1116 line. This may be due to the previously observed increase in lipid peroxidation in the RKO line. Insignificant changes in enzyme activity may therefore suggest that the tested compounds have no effect on GPx-related processes in colorectal cancer cells.

Catalase is an exosomal enzyme primarily responsible for converting hydrogen peroxide to water and molecular oxygen [37]. Depending on the colorectal cancer staging, a decrease or increase in catalase levels is observed [38]. In a study conducted by Piecuch et al. [38] on tissue sections taken from patients at different stages of CRC using immunohistochemical techniques, they observed differences in CAT expression. They concluded that in the case of adenoma, the level is increased, while in the case of adenocarcinoma, an increase in expression is observed. On the other hand, in a study by Finch et al. [39] conducted on an in vitro model of squamous cell carcinoma line 6M90, they showed that a decrease in CAT activity contributes to increased malignancy, while an increase is correlated with inhibition of neoplastic cell proliferation associated with EFGR [39]. In the case of our study on colorectal cancer cell lines, we observed a decrease in CAT enzyme activity after administration of EB5 and cisplatin compared to betulin, which caused a slight increase in CAT activity in the case of the RKO line. Thus, this suggests that only betulin may have an effect on the inhibition of this enzyme activity. However, in the case of the SW1116 line derived from adenocarcinoma, an increase in CAT activity was observed after administration of 5FU and cisplatin, suggesting that these compounds may contribute to a greater extent to the inhibition of neoplastic cell proliferation.

The final step was to assess changes in the expression of *SOD1*, *SOD2*, *CAT* and *GPX3* genes in response to the tested compounds in the RKO and SW1116 cell lines. Depending on the tested compound and the cell line, we observed a different expression profile of the tested genes. The *GPX3* gene mRNA expression in the tested cell lines was not detected both in untreated and treated cells. Hu et al. [40] showed expression of the GPX3 gene in two colorectal cancer cell lines, Lovo and SW480 [40]. However, Pelosof et al. [41] reported that *GPX3* gene expression depends on colorectal cell line and the methylation state of the promoter region [41]. Our results show that in SW1116 and RKO cell lines, the expression of this gene was silenced, and tested compounds had no stimulative effect on it. The positive detection of GPx enzyme activity may result from the presence of isoenzymes other than *GPX3* in studied cell lines.

In the case of the *SOD1* gene, which encodes a protein localized in the cytoplasm, a statistically significant decrease in mRNA expression was observed in the SW1116 line after betulin treatment, which was not observed in the RKO line. This suggests that the SW1116 line is more sensitive to betulin, which may also result in reduced production of the enzyme upon prolonged exposure and thus cause oxidative stress and ROS accumulation in the neoplastic cells, causing cell death. A similar study was also conducted by Skrzycki et al. [42], who in turn studied the effect of culture conditions on the level of *SOD1* gene expression [42]. They found that under hypoxia conditions, *SOD1* levels drop, which translates into conditions in intestinal cancer tumors caused by rapid proliferation and less oxygen access to tumor cells. Hence, in in vitro studies examining oxidative stress, the atmospheric conditions of cultured cells should also be taken into account.

The product of the *SOD2* gene, in turn, is closely associated with mitochondrial localization [43]. In our study, we observed that the EB5 derivative reduces *SOD2* expression in RKO and SW1116 cells, and in the case of the SW1116 line, reduced *SOD2* expression was also observed after betulin treatment. Consequently, it can be assumed that the tested compounds have a higher affinity for *SOD2* than *SOD1*, and thus, with prolonged exposition, a lower amount of the protein can be expected, which translates into reduced antioxidant capacity of colorectal cancer cells and associated cell death. Zhou et al. [44] conducted a study on DLD-1 and SW480 colon cancer cell lines, which they introduced using shRNA knock-out of the *SOD2* gene in order to decrease its role [44]. They showed that deletion of the gene encoding SOD2 results in a decrease in its expression and thus in the migration and proliferation of cancer cells [44]. In addition, they showed that inhibition of *SOD2* causes mitochondrial dysfunction and inhibits phosphorylation of the AMPK pathway, responsible for the survival of cancer cells [44]. Thus, it follows that these compounds may act by affecting AMPK phosphorylation, indirectly leading to CRC cell death, but this requires further study.

At the mRNA level, we also evaluate the *CAT* gene, which encodes catalase. We observed a decrease in *CAT* gene expression in the RKO and SW1116 cell lines after 5FU treatment, and additionally in the case of SW1116 upon exposure to betulin. The team of Giginis et al. [45] conducted a similar study examining the effect of knock-out using the CRISPR/Cas9 system of the *CAT* gene in the PC3 prostate cancer line [45]. They showed that reducing the level of expression of the gene encoding catalase contributes to the inhibition of the growth of neoplastic cells and also contributes to the loss of adhesion in the case of neoplastic cells. Therefore, it can be speculated that tested compounds may affect the processes involved in colorectal cancer prognosis.

Summarizing the results of our study, we can conclude that betulin and its derivative EB5 influence the level of antioxidant status in colorectal cancer cell lines RKO and SW1116. It can be noted that these cell lines react differently to the tested compounds, which is primarily due to their genetic and epigenetic differences. For this reason, in order to indicate new potential therapeutic substances, one should also take into account the differences between cell lines and the fact that the conditions of an in vitro effect may not be equivalent to in vivo conditions.

## 5. Conclusions

Studies have shown that betulin and its derivatives affect the viability of colorectal cancer cells and their antioxidant system. The cytotoxicity effect of the tested compounds closely depends on the cell line; however, in the case of the ECH147 derivative, the effect is the greatest. The RKO and SW1116 cell lines show a different sensitivity to the tested compounds at a concentration of 10 µg/mL, possibly due to their varying genetic and epigenetic profiles. Betulin and its derivative EB5 exhibit altered effects on redox homeostasis in the RKO and SW1116 cell lines, which may suggest that cells at a later stage of the malignancy may have a stronger total antioxidant capacity. A future research direction could be the formulation of betulin and its derivatives to increase its bioavailability, as well as assessing its impact on the efficiency of delivery of the medicinal substance. Further steps may also be to evaluate its in vivo activity on an animal model, mimicking the conditions in the body more closely.

## Figures and Tables

**Table 1:** IC_50_ values for the tested lines and compounds.

	IC_50_ Value for Each Compound											
Cell Line	Betulin		ECH147		EB5		TR50		Cisplatin		5FU	
	µg/mL	µM	µg/mL	µM	µg/mL	µM	µg/mL	µM	µg/mL	µM	µg/mL	µM
RKO	11.16	25.20	1.18	2.04	12.16	24.58	4.33	6.29	6.35	21.17	>100	>769
HT29	8.49	20.09	0.63	1.09	8.25	16.68	10.28	14.95	>100	>333	>100	>769
DLD-1	4.29	10.15	0.69	1.19	11.08	22.40	7.87	11.44	27.72	92.39	>100	>769
HCT 116	4.63	10.96	3.24	5.59	16.67	33.70	6.71	9.76	3.02	10.05	>100	>769
Caco2	11.20	26.50	1.40	2.43	16.91	34.17	12.97	18.85	95.07	316.84	>100	>769
SW1116	9.77	23.12	5.21	8.99	34.40	69.53	33.12	48.14	>100	>333	>100	>769
CCD-841CoN	5.26	12.44	0.12	0.20	>100	>202	18.30	26.61	27.59	91.94	>100	>769

ECH147—29-diethoxyphosphoryl-28-propynylobetulin; EB5—28-propynylobetulin; TR50—28-betulinyl (diethoxyphosphoryl)methyl)-1H-1,2,3-triazole-4-carboxylate; 5FU—5-fluorouracil.

## Data Availability

Available on request and with regulations.

## Supplementary Materials

The following supporting information can be downloaded at https://www.mdpi.com/article/10.3390/cells13161368/s1. Figure S1A: ^1^H NMR, compound TR50 in the range of 0.7–2.53 ppm; Figure S1B: ^1^H NMR, compound TR50 in the range of 3.19–8.30 ppm; Figure S2: ^13^C NMR, compound TR50; Figure S3: ^31^P NMR, compound TR50; Figure S4: IR, compound TR50; Figure S5: HRMS (ESI), compound TR50.
