# Supplementary material for: The Influence of Betulin and Its Derivatives on Selected Colorectal Cancer Cell Lines’ Viability and Their Antioxidant Systems
Source: Cells. 2024 Aug 17;13(16):1368. doi: 10.3390/cells13161368 (PMC11352258; doi:10.3390/cells13161368)
Supplement: Supplementary file 1 [file cells-13-01368-s001.zip › cells-3110395-Supplementary Material.pdf]

Supplementary materials

The Influence of Betulin and Its Derivatives on Selected
Colorectal Cancer Cell Lines' Viability and Their Antioxidant Systems

**Marcel Madej [1,*], Celina Kruszniewska-Rajs [1], Magdalena Kimsa-Dudek [2], Agnieszka Synowiec-Wojtarowicz [2], Elwira Chrobak [3], Ewa Bębenek [3], Stanisław Boryczka [3], Stanisław Głuszek [4,5], Jolanta Adamska [1],
Sebastian Kubica[1], Jarosław Matykiewicz [4,5] and Joanna Magdalena Gola [1,*]**

1 Department of Molecular Biology, Faculty of Pharmaceutical Sciences in Sosnowiec, Medical University of Silesia, 40-055 Katowice, Poland; ckruszniewska@sum.edu.pl (C.K.-R.); jolaa@sum.edu.pl (J.A.); sebastian.kubica@sum.edu.pl (S.K.)

2 Department of Nutrigenomics and Bromatology, Faculty of Pharmaceutical Sciences in Sosnowiec, Medical University of Silesia, 40-055 Katowice, Poland; mkimsa@sum.edu.pl (M.K.-D.); asynowiec@sum.edu.pl (A.S.-W.)

3 Department of Organic Chemistry, Faculty of Pharmaceutical Sciences in Sosnowiec, Medical University of Silesia, 40-055 Katowice, Poland; echrobak@sum.edu.pl (E.C.); ebebenek@sum.edu.pl (E.B.); boryczka@sum.edu.pl (S.B.)

4 Department of Surgical Medicine with the Laboratory of Medical Genetics, Institute of Medical Sciences, Collegium Medicum, Jan Kochanowski University, 25-317 Kielce, Poland; sgluszek@wp.pl (S.G.); jaroslaw.matykiewicz@onkol.kielce.pl (J.M.)

5 Department of Clinic Oncological Surgery Holycross Center, 25-317 Kielce, Poland

* Correspondence: marcel.madej@sum.edu.pl (M.M.); jgola@sum.edu.pl (J.M.G.); Tel.: +48-32-364-10-52 (M.M.); +48-32-364-12-34 (J.M.G.)

LIST OF CONTENTS

Experimental

Synthesis of compound TR50

Figure S1A. ^{1}H NMR spectrum of compound TR50 in the range of 0.7-2.53 ppm

Figure S1B. ^{1}H NMR spectrum of compound TR50 in the range of 3.19-8.30 ppm

Figure S2. ^{13}C NMR, compound TR50

Figure S3. ^{31}P NMR, compound TR50

Figure S4. IR, compound TR50

Figure S5. HRMS (ESI), compound TR50

Experimental

Synthesis of compound TR50

28-O-Propynoylbetulin (EB5) (0.1 g, 0.203 mmol), copper(I) iodide (0.004 g, 0.020 mmol) and 3.5 ml of dry toluene were placed in a round-bottom flask. Then 0.213 mmol of diethyl (azidomethyl)phosphonate was added. The reaction mixture was stirred at 50°C for 72 hours. The course of the reaction was monitored using TLC chromatography. The spots in the chromatogram were visualized by spraying with a 10% solution of sulfuric acid in ethanol and then heated at 110°C for 3 minutes. After evaporating the solvent from the reaction mixture, the obtained crude product was purified by column chromatography using a mixture of chloroform : ethanol (15:1 v/v).

Compound **TR50**

Yield 75%; mp 115-117°C; R_f 0.43 (chloroform/ethanol, 15:1, v/v);
^{1}H NMR (600 MHz, $CDCl_3$) δ: 0.78 (s, 3H, CH_3), 0.85 (s, 3H, CH_3), 0.99 (s, 3H, CH_3), 1.02 (s, 3H, CH_3), 1.08 (s, 3H, CH_3), 1.34 (2 x t, 6H, 2 x CH_3), 1.72 (s, 3H, CH_3), 0.69-2.07 (m, 26H, CH, CH_2 from the basic lupane system) 2.52 (m, 1H, H-19), 3.21 (m, 1H, H-3), 4.18 (m, 5H, 2x OCH_2, H-28), 4.59 (m, 1H, H-28), 4.73 (bs, 1H, H-29), 4.84 (bs, 1H, H-29), 4.90 (d, J = 13.2 Hz, 2H, NCH_2), 8.30 (s, 1H, C-H triazol); ^{13}C NMR (150 MHz, $CDCl_3$) δ: 14.8, 15.4, 16.06, 16.1, 16.3, 16.4, 18.3, 19.1, 20.8, 25.2, 27.1, 27.4, 28.0, 29.6, 29.8, 34.2, 34.7, 37.2, 37.7, 38.7, 38.9, 40.9, 42.8, 45.5, 46.6, 46.7, 47.7, 48.9, 50.4, 55.3, 63.6, 63.7, 63.8, 80.0, 110.0, 128.2, 140.67, 150.1, 160.9; IR (ν max cm^{-1}, KBr): 1244-1201, 1456, 1544, 1724, 2945, 3442; ^{31}P-NMR (243 MHz, $CDCl_3$) δ: 14.97; HRMS (ESI) m/z (positive): $C_{38}H_{63}N_3O_6P$ $[M+H]^+$ Calculated 688.4454, Found 688.4458; $C_{38}H_{62}N_3O_6PNa$ $[M+Na]^+$ Calculated 710.4274, Found 710.4279; $C_{38}H_{62}N_3O_6PK$ $[M+K]^+$ Calculated 726.4013, Found 726.4021.

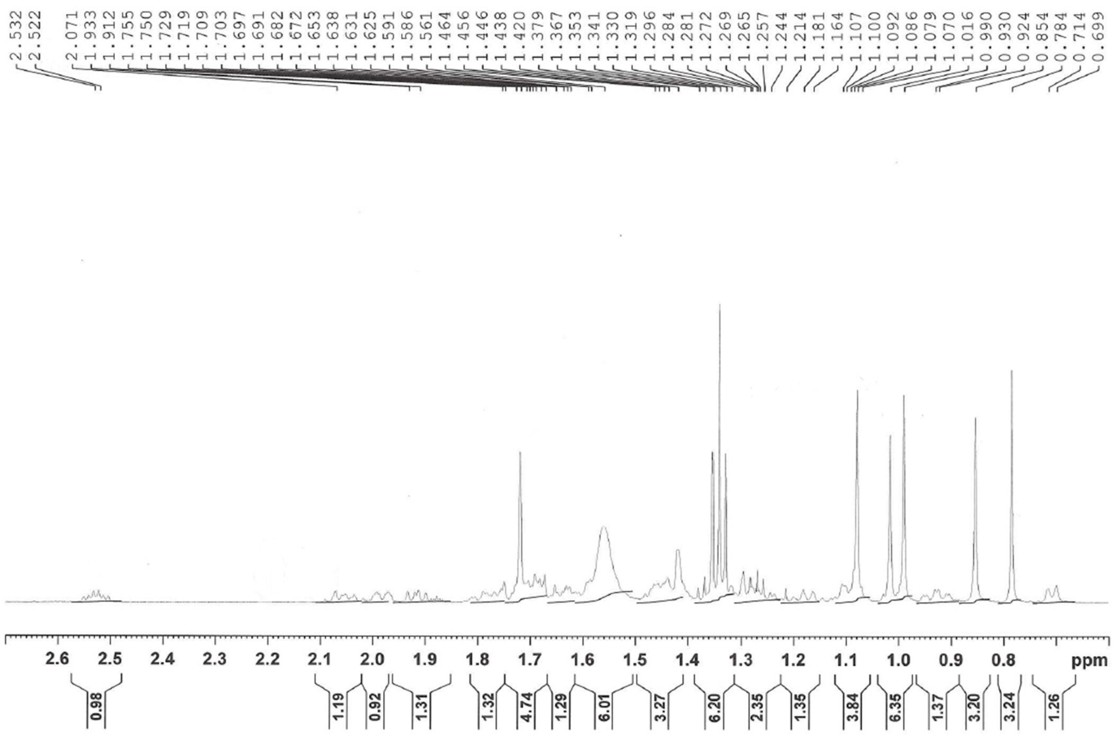

Figure S1A. ^{1}H NMR spectrum of compound TR50 in the range of 0.7-2.53 ppm.

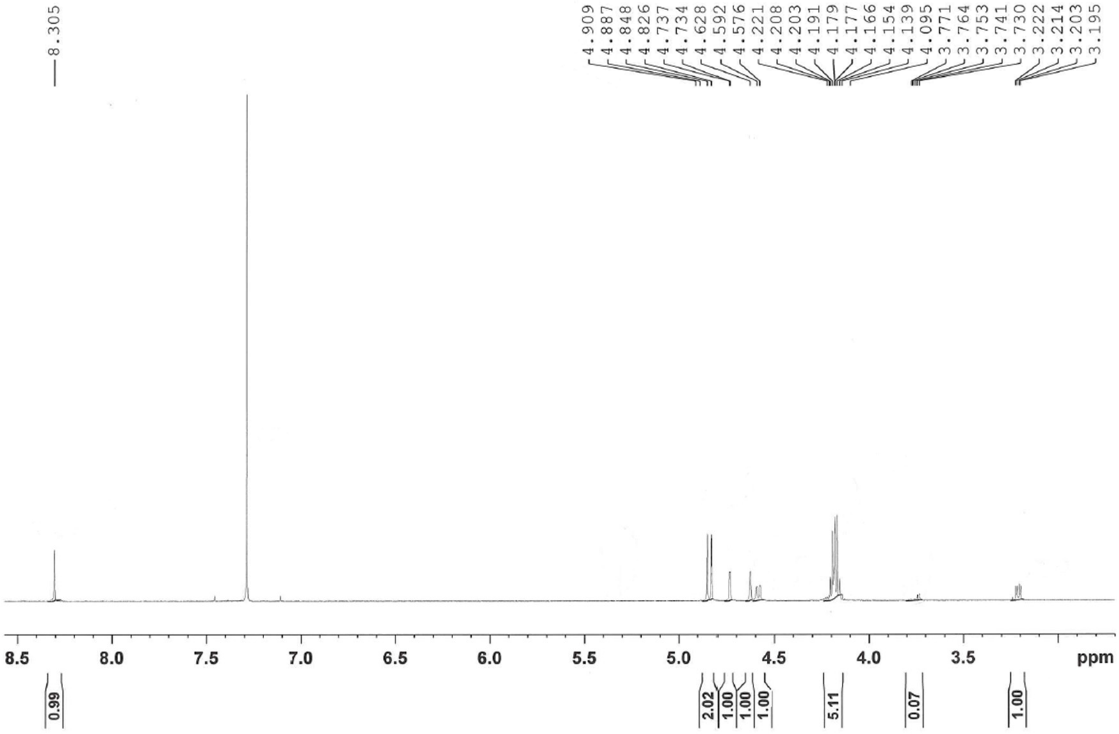

Figure S1B. ^{1}H NMR spectrum of compound TR50 in the range of 3.19-8.30 ppm.

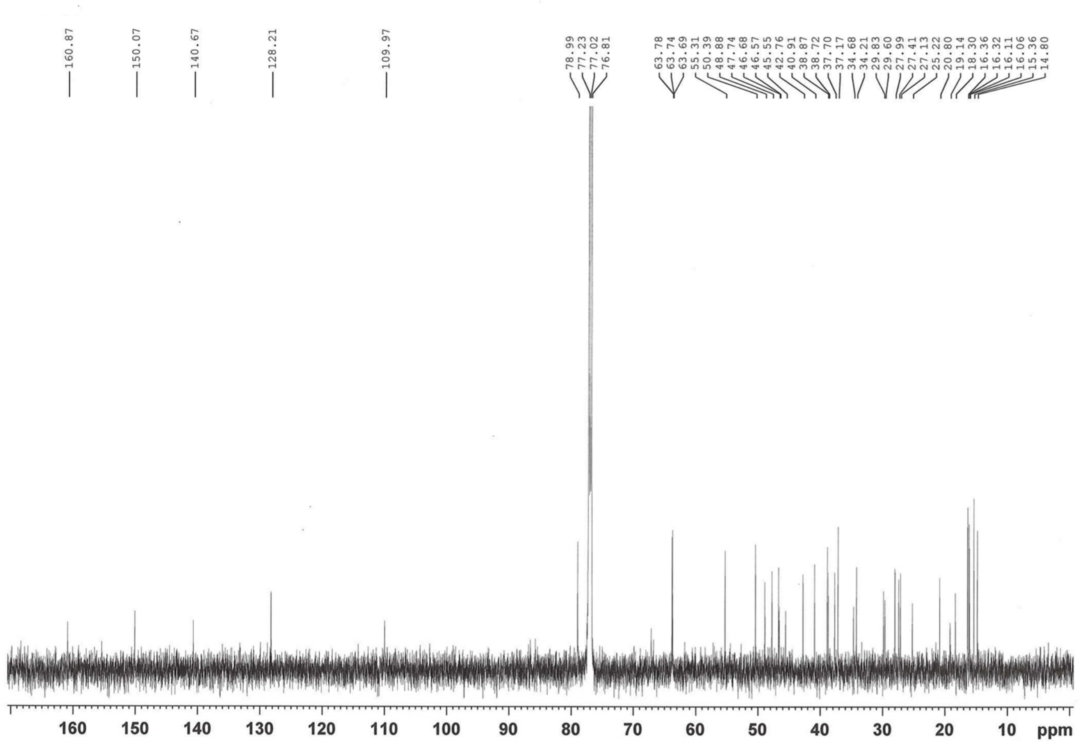

Figure S2. ^{13}C NMR, compound TR50.

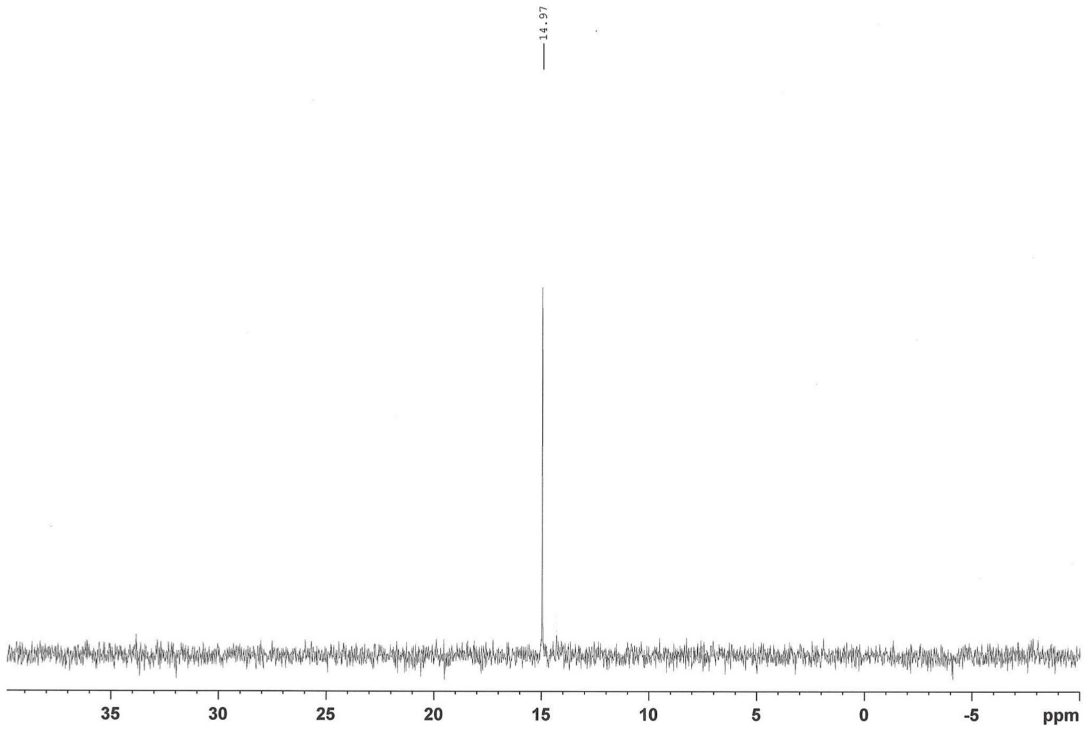

Figure S3. ^{31}P NMR, compound TR50.

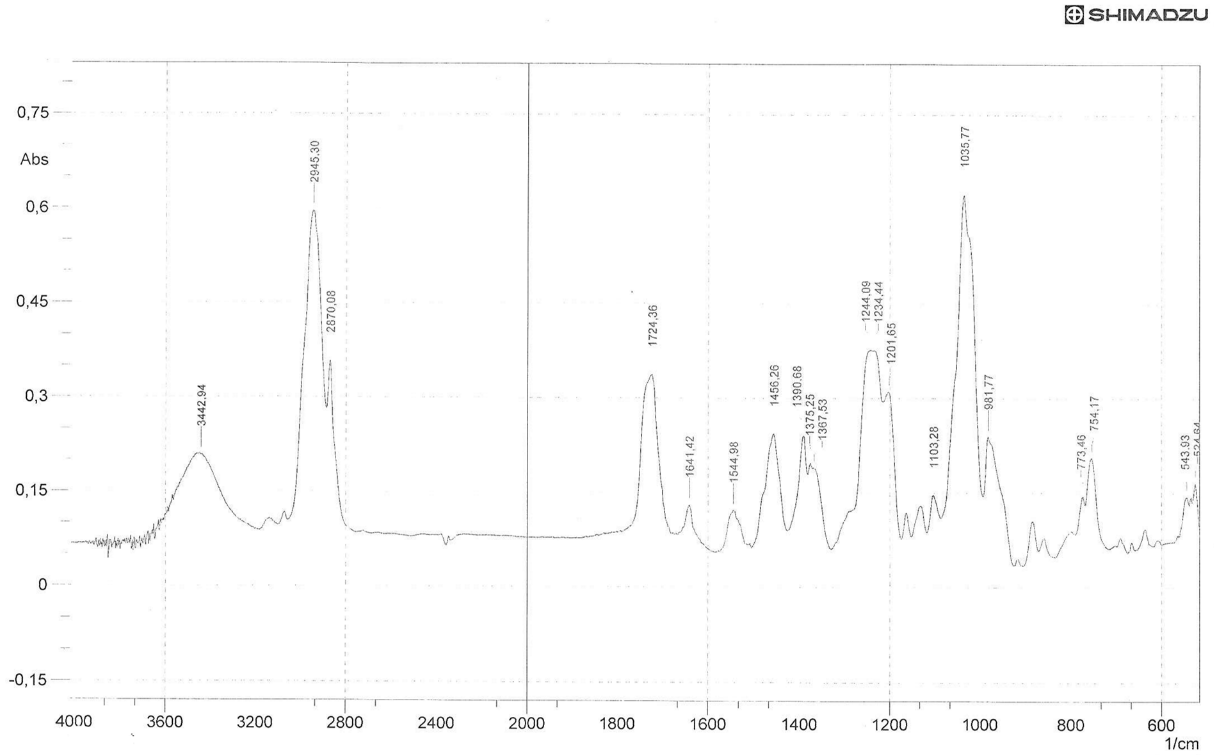

Figure S4. IR, compound TR50.

Mass Spectrum List Report

Analysis Info

Analysis Name D:\Data\TR50.d
Method low_mass_positive.m
Sample Name cf3
Comment

Acquisition Date 12/8/2023 12:57:46 PM

Operator KM
Instrument impact II 1825265.10082

Acquisition Parameter

Source Type	ESI	Ion Polarity	Positive	Set Nebulizer	0.3 Bar
Focus	Active	Set Capillary	4000 V	Set Dry Heater	200 °C
Scan Begin	100 m/z	Set End Plate Offset	-500 V	Set Dry Gas	3.0 l/min
Scan End	1000 m/z	Set Charging Voltage	2000 V	Set Divert Valve	Source
		Set Corona	0 nA	Set APCI Heater	0 °C

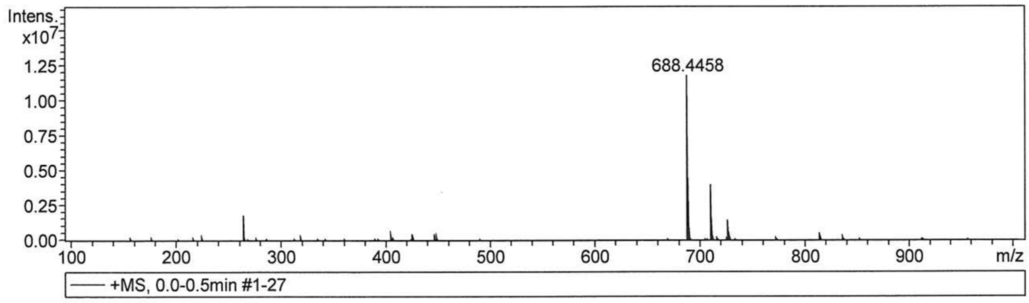

#	m/z	Res.	S/N	I	I %	FWHM
1	688.4458	53202	24553.9	11720679	100.0	0.0129
2	710.4279	45292	8262.0	4041695	34.5	0.0157
3	726.4021	35844	2950.8	1462067	12.5	0.0203

Figure S5. HRMS (ESI), compound TR50.
